# Diagnosis and Management of Osteoporosis During COVID-19: Systematic Review and Practical Guidance

**DOI:** 10.1007/s00223-021-00858-9

**Published:** 2021-05-18

**Authors:** G. Hampson, M. Stone, J. R. Lindsay, R. K. Crowley, S. H. Ralston

**Affiliations:** 1 Department of Chemical Pathology and Metabolic Medicine, St Thomas’ Hospital, Lambeth Palace Road, 5th Floor, North Wing, London, SE1 7EH UK; 2 Department of Rheumatology, Metabolic Bone Clinic, Guy’s Hospital, London, UK; 3 Metabolic Bone Service, University Hospital Llandough, Llandough, Penarth CF64 2XX UK; 4 Osteoporosis and Bone Metabolism Service, Musgrave Park Hospital, Belfast, Northern Ireland UK; 5 Department of Endocrinology, St Vincent’s University Hospital, Dublin, Ireland; 6 University College Dublin, Dublin, Ireland; 7 Centre for Genomic and Experimental Medicine, Institute of Genetics and Cancer, University of Edinburgh, Western General Hospital, Edinburgh, EH4 2XU UK; 8 Rheumatic Diseases Unit, NHS Lothian Western General Hospital Edinburgh, Edinburgh, EH4 2XU UK

**Keywords:** COVID-19, Osteoporosis, FRAX, Osteoporosis management

## Abstract

It is acknowledged that the COVID-19 pandemic has caused profound disruption to the delivery of healthcare services globally. This has affected the management of many long-term conditions including osteoporosis as resources are diverted to cover urgent care. Osteoporosis is a public health concern worldwide and treatment is required for the prevention of further bone loss, deterioration of skeletal micro-architecture, and fragility fractures. This review provides information on how the COVID-19 pandemic has impacted the diagnosis and management of osteoporosis. We also provide clinical recommendations on the adaptation of care pathways based on experience from five referral centres to ensure that patients with osteoporosis are still treated and to reduce the risk of fractures both for the individual patient and on a societal basis. We address the use of the FRAX tool for risk stratification and initiation of osteoporosis treatment and discuss the potential adaptations to treatment pathways in view of limitations on the availability of DXA. We focus on the issues surrounding initiation and maintenance of treatment for patients on parenteral therapies such as zoledronate, denosumab, teriparatide, and romosozumab during the pandemic. The design of these innovative care pathways for the management of patients with osteoporosis may also provide a platform for future improvement to osteoporosis services when routine clinical care resumes.

**Supplementary Information:**

The online version contains supplementary material available at 10.1007/s00223-021-00858-9.

## Introduction

The rapid spread of infection caused by the severe acute respiratory syndrome coronavirus 2 (SARS-CoV-2) was categorised by the World Health Organisation (WHO) as a pandemic in March 2020. As of January 2021, over 90 million cases have been confirmed worldwide with over 1.9 million deaths [1]. In the UK, over 4.1 million people have tested positive with in excess of 122,000 deaths as of February 2021 [1]. In most young people SARS-CoV-2 is asymptomatic or causes mild influenza-like symptoms with loss of taste and smell [2]. However, others may develop a life-threatening illness with acute respiratory distress syndrome (ARDS) and multiorgan failure requiring hospital admission and ventilatory support [3]. Severe forms of the illness are associated with activation of the immune system, with increased production of pro-inflammatory cytokines and raised levels of CRP [4]. The risks of hospital admission and poor outcome increase markedly with age and are associated with pre-existing obesity, hypertension, cardiovascular disease, and ethnic group. Since the first reported cases of the disease in Wuhan, China in December 2019, a series of measures have been implemented worldwide to limit the spread of the virus including travel bans, limits on public gatherings and nationwide lockdowns. Public health approaches have included social distancing and infection control measures such as frequent hand washing, sanitisation and more recently vaccinations. Introduction of these strategies, has been partially successful in mitigating virus spread but has caused severe disruption to healthcare and social services [5]. Because of the prioritisation of urgent services and delaying of elective care, the management of many chronic or long-term medical conditions, such as osteoporosis, has been challenging as resources are diverted from chronic diseases care to combat the pandemic [6]. The potential impact of this is considerable since osteoporosis is the commonest bone disease worldwide affecting 1 in 2 women and 1 in 5 men at some point in life. It is estimated that the cost of osteoporosis is 37 billion EUR per year in the EU, and 19 billion USD per year in the USA due, in part, to hospitalisation as a result of fractures [7]. Costs are projected to rise dramatically alongside an increasing osteoporosis prevalence in coming years with a larger elderly population and it is estimated that osteoporotic fractures cause an annual global loss of 5.8 million healthy life years to disability and reduced relative survival. Hip fractures are associated with a 30% mortality rate at 1 year and 53% of patients who sustain a hip fracture are no longer able to live independently [8]. Here we have conducted a systematic review of the literature to assess the impact of the pandemic on the diagnosis and treatment of osteoporosis and documented the adaptations that have been made in osteoporosis services in five secondary referral centres in the supplementary material.

## Methods

**Fig. 1:**
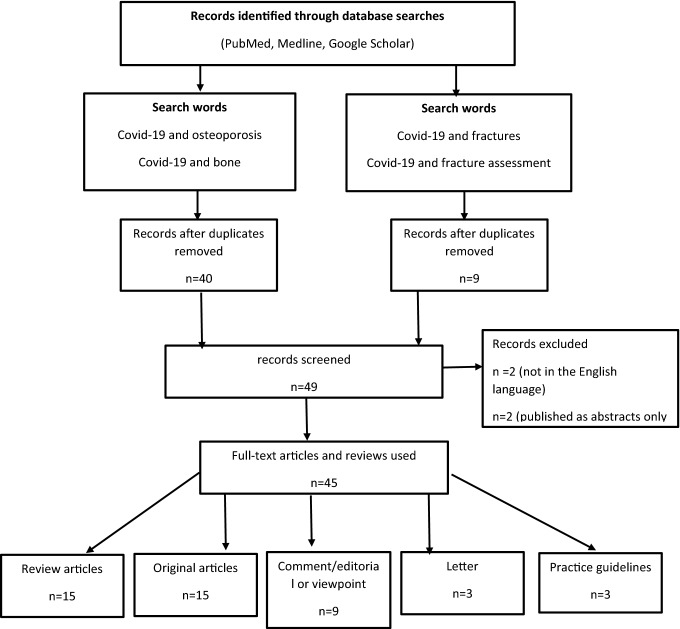
PRISMA flow diagram of the search for eligible studies COVID-19 and osteoporosis or fractures

A literature review of electronic databases (PubMed, Medline, Google Scholar) was conducted by one of the authors including the following words ‘COVID-19 and osteoporosis or bone or COVID-19 and fractures or fracture assessment’ for the selection of studies that described the effect of the COVID-19 pandemic on fracture liaison and osteoporosis services including osteoporosis diagnosis, clinical management, and outcomes where evidence was available. The search was carried out from January 2020 to February 2021. The identification of relevant articles including review articles, practice guidelines, original articles, comment/editorial/viewpoint, letters was performed by GH. The main source of information was obtained from published articles. Criteria for inclusion were (1) written in English language, (2) publications reporting the impact of COVID-19 on bone health. The search using the terms ‘COVID-19 and osteoporosis’ or ‘COVID-19 and bone’ yielded 40 publications after duplicates were removed. Two were not written in English and a further two were published in abstract form only and were excluded. Thirty-six were reviewed and comprised of practice guidelines (*n* = 3), review articles (*n* = 13), original articles (*n* = 8), viewpoint/editorial (*n* = 9), letter (*n* = 3). Using the terms COVID-19 and fractures identified a further nine publications (7 original articles and 2 review articles). The selection of studies is shown in a PRISMA flow diagram (Fig. 1).

### Fracture Risk Assessment

The two key steps in risk assessment of patients suspected to have osteoporosis is a fracture risk assessment which may also be coupled to a measurement of bone mineral density (BMD) [9, 10]. There are various means of fracture risk assessment [11, 12] but one of the most commonly used is the FRAX calculator which is available for 66 countries covering 80% of the world population. It has been adopted in several country-specific guidelines for initiation of treatment to reduce fractures [13–15]. Although FRAX can be calculated on the basis of clinical risk factors alone and does not necessarily require attendance at hospital for a BMD measurement, a study by McCloskey and colleagues showed that the use of FRAX was significantly reduced over the 3-month period from February 2020 to April 2020 [16]. The number of sessions, defined as FRAX tool usage within a 30-min time frame, fell by 23% and 58% in March and April 2020 respectively compared to the same period in 2019. In Europe, the majority of countries reduced their usage by at least 50%. In Latin America, the reductions were higher than 50% with smaller reductions seen in Asian countries. The authors estimated that over the 3-month period, approximately 500,000 fewer individuals were assessed for their risk of fracture than normal [16]. The reasons for this were not explored in the study but our experience suggests that this may have been due to difficulties in patients attending their GP’s and/or being referred to hospital clinics for these calculations due to diversion to resources from elective care to urgent care as the result of the pandemic.

### Bone Density Measurements

Measurement of bone mineral density (BMD) by dual-energy X-ray absorptiometry (DXA) is a valuable tool for the diagnosis of osteoporosis, for improving the precision of fracture risk assessment following the use of screening tools like FRAX and for monitoring the response to osteoporosis therapy. The evidence which supports the use of osteoporosis treatments to reduce fracture risk was gained by randomised controlled trials of individuals who had low BMD values on DEXA and/or low trauma vertebral fractures [10]. Several studies have also shown that change in BMD following treatment is correlated with a reduction in fracture risk [17]. Measurements of BMD are largely provided by secondary-care facilities in the UK and this has been compromised and disrupted during the pandemic [18]. Diagnostic imaging or radiological procedures have been prioritised during the pandemic based on clinical urgency and in many centres DXA services have been deprioritised or even paused temporarily. In centres where DXA service have continued to operate the throughput has markedly diminished due to stringent infection control measures and social distancing. In addition, frail and elderly patients who are particularly susceptible to fragility fractures associated with osteoporosis have been reluctant to attend hospital for fear of contracting SARS-CoV2. Accordingly, across the UK it was estimated that there were 73% fewer DXA scans carried out in June 2020 compared to 2019 [18]. As a result of this, it is almost certain that many months may pass before the backlog is cleared and waiting lists return to acceptable levels.

### Biochemical Investigations

In some centres, issues have been experienced with the availability of laboratory tests including serum calcium, creatinine, and 25(OH) D which may be measured as part of safety checks before treatment with zoledronic acid or denosumab. Biochemical markers of bone turnover such as PINP are also used in some centres to monitor adherence to treatment. Social distancing measures as well as the reduction in access to phlebotomy during the pandemic has meant that it has often been challenging to have these pre-treatment blood tests performed. Strategies that are being explored to surmount these issues are the establishment of “mobile” phlebotomy vans and also engagement with local pharmacies to offer phlebotomy services where GP’s are finding it difficult to provide phlebotomy services.

### Fracture Liaison Services

Fracture liaison services (FLS) are structured secondary prevention programmes for men and women aged 50 years or older after a fragility fracture [19]. These involve a multi-disciplinary diagnostic-therapeutic pathway for the management of complications in patients with a recent fragility fracture with the aim of reducing falls and /or a new fragility fracture and ensures appropriate care and treatment in high-risk patients after discharge from hospital [20]. These patients are identified during their attendance at a fracture clinic or hospitalisation after an acute fragility fracture, often through scrutiny of the electronic patient record. Typically, those with major osteoporotic fractures aged > 50 years are offered DXA and, depending on the results, initiation of osteoporosis treatment is recommended. Some guidelines recommend initiation of treatment in all patients with a fracture above a certain age without recourse to a DXA. The FLS have been shown worldwide to be effective clinically in reducing the risk of fractures after a first fracture [21]. In the current pandemic, many FLS including the associated rehabilitation services have largely closed as resources are diverted to the care of patients with COVID-19. A recent survey of out-patient attendances to the fracture clinic for non-hip fragility fractures in a large university hospital showed a decline during lockdown to a mean number of 26.0 (SD 7.3) from 63.1 (SD 12.6) outpatients per week prior to lockdown in 2020 and in previous years. It has been speculated that the reduction in non-hip fractures may be due in part to fewer falls as a result of movement restrictions [22]. Some centres, however, have offered virtual fracture liaison clinics during the COVID-19 pandemic leading to reduced delays in the initiation of fracture prevention therapies [23].

### Care of Patients with Hip Fracture

Data on the rates of hip fracture have been conflicting during the pandemic such that some studies have reported no change compared with previous years [22], whereas others have described increases or decreases [24–26]. Delays to surgery due to limited theatre access have been reported as surgical wards have been reconfigured to cope with increasing numbers of admissions related to COVID-19 [26]. In addition, hip fracture patients may not be given adequate care following discharge through redeployment of key staff such as physiotherapists and occupational therapists to acute services. A retrospective audit highlighted a significant reduction in the review of patients with femoral neck fractures by the orthogeriatric team after the lockdown due to redeployment of staff [27]. Reductions in the prescription for calcium/vitamin D supplements and osteoporosis medications were seen at the same time which may have a significant impact on fracture burden with resultant increases in morbidity and mortality [27].

### Medications for Osteoporosis

Globally, there is a documented treatment gap in the management of osteoporosis as only one-fifth of patients have been estimated to receive appropriate treatment after a hip fracture at a time where they are at highest risk of another fracture [28]. The COVID-19 pandemic has further exacerbated this gap as treatment of patients with osteoporosis is considered low on the list of clinical priorities, including zoledronic acid (ZA) which is usually delivered in hospital day units, and teriparatide and romosozumab which are typically initiated by specialists in secondary care. Denosumab can be initiated in primary care but in the UK and the ROI, treatment is usually commenced on the recommendation of an osteoporosis specialist in secondary care, although this may change in the ROI.

Capacity for delivery of infusion treatments has been reduced due to redeployment of staff to acute services, and reduced capacity for delivery of infusions due to social distancing. Finally, many patients have been unwilling or unable to attend secondary care for scheduled treatments. With ZA the timing of the next dose is not critical as its anti-resorptive effect is sustained for up to 2 years following an infusion due to its long skeletal retention time [29]. Reflecting this fact, it has been demonstrated that there is prolonged protecting against fractures after treatment with zoledronic acid [30, 31] and other oral bisphosphonates, [32, 33]. Accordingly, even if a scheduled infusion needs to be delayed for several months this is unlikely to be harmful.

Denosumab presents specific problems since its inhibitory effects on bone resorption disappear quickly when the administration is delayed beyond 7 months after the last dose [34, 35]. Furthermore, it is now recognised that patients who stop denosumab have a rebound increase in bone remodelling for 6–12 months after stopping therapy, and this can be associated with the occurrence of multiple vertebral fractures and even hypercalcaemia [36]. Accordingly, current guidelines recommend that the delay in denosumab should not exceed 1 month from the scheduled date of injection [35, 36] Discontinuation of teriparatide (TPTD) also leads to bone loss over the first 12 months, but there is no evidence of a rebound increase in bone remodelling or an increased risk of vertebral fractures [37]. Even if TPTD needed to be stopped due to interruptions of supply, bone loss could be mitigated by prescription of an oral bisphosphonate which has been shown to maintain the increase in BMD and protect against fractures for up to 5 years [38, 39]. Discontinuing romosozumab also leads to bone loss within 12 months and there is evidence of increased bone resorption within 3 months of stopping, although there is insufficient evidence to ascertain whether this leads to increased risk of rebound fractures [40]. If romosozumab needs to be stopped for any reason bone loss could be prevented with an antiresorptive drug such as an oral bisphosphonate.

### Does Osteoporosis Influence Outcome of SARS-Cov2 Infection?

A large number of clinical risk factors have been associated with mortality in patients with COVID-19 disease, the most important of which are age, BMI, ethnic group, respiratory disease, cardiovascular disease, chronic kidney disease, chronic liver disease, neurological disease, and immunosuppression [41]. An interesting observation from the QResearch database of 1205 GP practices in England was that a history of hip, spine, humerus, and wrist fractures was associated with an increased risk of death from SARS Cov2 infection in women (1.12, 95% CI, 1.00–1.260) and in men 1.35 [1.24–1.47]. Individuals with previous fractures also had an increased risk of hospital admission due to SARS Cov2 infection [42]. Another small study investigated the prevalence of morphometric vertebral fractures (VFs) among patients with SARS Cov2 infection. This study showed that VF’s were common (about 36%) among those hospitalised with severe SARS Cov2 compared with the general population where the prevalence of VFs ranged from 18–26% in women and 8–20% in men. The presence of VFs in this study was a strong prognostic marker and predictor of clinical outcomes and disease severity as other well-described risk factors and co-morbidities, although the prevalence of VFs in the study population was higher than previously reported in the European general populations [43, 44]. Furthermore, patients with VFs were more likely to require non-invasive mechanical ventilation (48.8% vs 27.4%, *p* = 0.02). Mortality was higher in those with severe VFs (60%) compared with those with moderate or mild VFs (7% and 24%, respectively) [43]. The author speculated that impaired respiratory function and kyphosis associated with VF’s may decrease vital lung capacity and increase the risk of severe SARS Cov2 infection. In a recent meta-analysis the prevalence of SARS Cov2 infection in hip fracture patients ranged from 1 to 28%, with a mean of 13% [45]. Data from 21 studies which reported mortality following hip fracture showed that COVID-19 positive patients have a seven-fold increased risk of death compared to COVID-19 negative patients. Crude mortality rate was 35% in those with COVID-19 infection compared to 8% in those without. However, the studies did not adjust for confounders such as age, sex, co-morbidities, level of independence, frailty which are known to be associated with mortality risk after a hip fracture. [45].

### Does SARS-Cov 2 Infection Predispose to Osteoporosis?

Since COVID-19 infection leads to increased pro-inflammatory cytokine production and can be associated with prolonged immobilisation in seriously ill patients, this might be expected to increase bone resorption and promote bone loss [4].

In addition, the medium and long-term sequelae of the infection may be expected to have a negative impact on the skeleton. Evidence shows patients who have suffered severe SARS Cov2 continue to experience health problems including breathing difficulties, cardiovascular problems, loss of muscle mass, muscular weakness, mobility issues, and impaired activities of daily living [46]. Thus, people suffering from the medium to long-term effects of COVID-19 infection will need a comprehensive recovery and rehabilitation treatment plan to tackle these adverse outcomes. Where appropriate, older individuals recovering from SARS Cov2 may need to undergo a fracture risk assessment and DXA coupled to anti-osteoporosis treatment [47].

In addition, the measures implemented during the pandemic, such as the travel bans, quarantines, self-isolation has led to reduced physical activity, particularly in the elderly population predisposing to loss of muscle mass and function and contributing to sarcopenia [48].

### Managing Osteoporosis During Pandemic

As reviewed above, the pandemic has presented several challenges for treating osteoporosis, particularly in patients on parenteral therapies. The concerns surround the establishment and implementation of effective alternative strategies, and the adoption of new ways of practice such as remote consultations to reduce fracture burden has been proposed [49]. There is evidence of the use of telemedicine approaches in the field of osteoporosis. A study from Canada comprising of a mailed satisfaction survey and telephone interviews to understand patient experiences of osteoporosis care delivered virtually by telemedicine showed that the patients’ perception of care by telemedicine was comparable to face-to-face visits with added benefits which included convenience, reduced travel time, and costs. Nevertheless, there was a need for improvement as patients were concerned about the follow-up with allied professionals such as physiotherapists and the co-ordination of investigations and tests. They also expressed interest in the design of an osteoporosis virtual self-management program focusing on advice about diet and lifestyle factors [50]. Follow-up telephone consultations to promote adherence already forms part of the FLS in the UK, although this has been disrupted in the current pandemic. However, the effectiveness of telephone consultations on adherence rates to osteoporosis medication use has been mixed with modest effect reported in a Canadian trial and no difference seen in two American studies [51–53].

Clinical guidance on screening and treatment developed and endorsed by the American Society for Bone and Mineral Research (ASBMR), Endocrine Society, the American Association of Clinical Endocrinologists, the European Calcified Tissue Society and the European Society for Endocrinology have been published [54]. These recommendations were largely based on expert opinion as evidence-based data were lacking.

The difficulty in accessing DXA scans during the pandemic has been highlighted previously in this review. An algorithm has been suggested based on the use of FRAX in patients who had been referred for DXA. The authors suggested that this could be done through a telephone consultation during which information about the patient’s age, gender, weight, height, clinical risk factors are obtained to derive the FRAX score. It was suggested that following calculation of FRAX, patients could be categorised as low risk, intermediate risk, and high risk. Subsequently, those in the intermediate-risk category can proceed with DXA examinations, those in the low-risk category can be reassured (or DXA deferred) and those in the high-risk treated without DXA [55]. This is very similar to the approach advocated in the NOGG guidance when DXA scans are unavailable or impractical. There are several potential issues with this approach, one is gaining accurate information on height, weight, and other risk factors; a second would be difficulties in communication with people who were hard of hearing and those with cognitive problems. As has been mentioned previously there is limited information on how effective treatments for osteoporosis are in patients with high fracture risk alone in the absence of information from DXA. The SCOOP study provided some insights into the effectiveness of treating individuals at high risk of fracture without DXA. During this 5-year study, 24% of individuals in the “screened” group received at least one prescription for anti-osteoporosis treatment compared with 16% of the control group. The hazard ratio for fracture was 0.94 [0.85–1.03] in the screened group a difference that was not significant. Hip fractures were less common in the screened group however (0.72 [0.59.0.89]) [56]. It should be noted that in SCOOP, the 10-year risk of hip fracture in both groups was about 7.5% which is considerably higher than the “treat” recommendation based on hip fracture probability in the NOGG algorithm.

The authors also suggested that the majority can be delayed based on clinical judgement, particularly if this is for monitoring patients who are on osteoporosis treatment or when previous scans have shown stable BMD and there are no new clinical risk factors [55]. It was suggested that priority should be given, however, to those who have had a significant decline in BMD on previous DXA scans or who develop new risk. While the algorithm is pragmatic the efficacy has not been tested in routine clinical care, and it is, therefore, unclear if it would reduce fracture burden in these times.

**Fig. 2:**
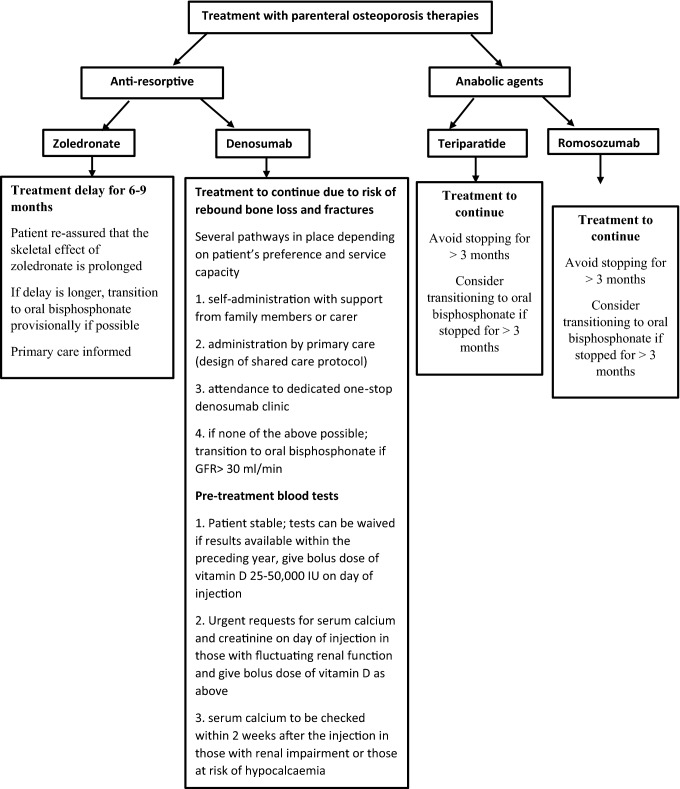
Algorithm summarising the guidance to the management of osteoporosis during the COVID-19 pandemic in patients on parenteral treatment. All patients should continue calcium and/or vitamin D supplements and be encouraged to maintain a healthy-balanced diet and lifestyle such as stopping smoking, avoiding excessive alcohol intake (> 3 units/day), keeping active and exercising regularly. Adapted from Yu, E. W. et al. Osteoporosis management in the era of COVID-19 [53] and Gittoes et al. Endocrinology in the time of COVID-19: management of calcium metabolic disorders and osteoporosis [56]

The joint guidelines of the bone health organisations addressed the management considerations, particularly those receiving treatment with iv bisphosphonates (zoledronate), denosumab, teriparatide, and romosozumab [54, 57] and are summarised in Fig. 2. There is no evidence that any of these therapeutic agents increases the risk of SARS-Cov-2 infection but a meta-analysis of 33 randomised controlled trials (22,253 patients) showed that denosumab is associated with an increased risk of severe infections, especially of ear, nose and throat infections [58]. A telephone survey of 85 patients on osteoporosis medications including denosumab attending the bone clinic in the Endocrine Division of San Raffaele Hospital Milan, one of the epicentres of COVID-19 pandemic in Italy, was undertaken between 21st February to 24th May 2020 [59]. Forty-two patients responded and 26 were on denosumab. Only 1 patient (3.8%) on denosumab reported symptoms consistent with a respiratory tract infection during the pandemic but did not have a COVID-19 swab test. None of the patients on denosumab were hospitalised. Although, it appears that treatment with Denosumab is not a specific risk factor for COVID-19 infection and data from this small ‘real-life’ study support the guidelines that denosumab should continue during the pandemic, larger studies may be needed to confirm this.

There is also no evidence that any of the osteoporosis therapies interferes the efficacy or side-effect profiles of the COVID-19 vaccines. However, some minor changes to the timing of the osteoporosis drug administration may be considered to account for the patient’s COVID-19 vaccine schedule [60].

### Maintenance and Recovery of Osteoporosis Services

Information about the adaptations to the metabolic bone/osteoporosis services in the authors' centres during the pandemic is provided in the supplementary material.

#### Lifestyle Measures

We continue to emphasise the importance of lifestyle measures such as maintaining a healthy balanced diet, stopping smoking and reducing alcohol intake (< 3 units/day) and taking regular exercise while acknowledging that this is more difficult due to restrictions on leaving home and closing of facilities like leisure centres and gyms. To mitigate this, home-based resistance exercise interventions aimed at increasing physical activity could be implemented through telehealth and interaction with a physiotherapist and could also include the use of educational exercise videos [48].

#### Vitamin D Supplements

Because of the fact that frail older individuals may be shielding and unable to get outside we endorse the advice for vitamin D supplementation and calcium where required if dietary intake is low. For patients receiving intravenous bisphosphonates higher dose supplements can be used such as a bolus doses of cholecalciferol (25–50,000 IU) a few days before treatment since these have been shown to increase serum 25 (OH) vitamin D concentrations within three days after oral supplementation [61]. Doses, however, probably should not exceed 50,000 IU as bolus doses of 500,000 IU annually and 60, 000 IU monthly were associated with increased risk of falls and fractures in populations who were not vitamin D deficient [62, 63].

Observational studies report an association between vitamin D deficiency and susceptibility to respiratory infections due to potential benefits on the immune system; data in the context of COVID-19 are inconsistent and evidence for causality is lacking [64, 65]. There are several ongoing trials investigating the effect of vitamin D supplementation on COVID-19 prevention and management which may help provide further guidance [64].

#### Intravenous Bisphosphonates

Due to the long half-life of bisphosphonates in the bone we advise that delaying infusions for time periods of up 12 months is unlikely to lead to increased fracture risk or significant decline in BMD as zoledronate is long-acting [30–33]. While transitioning to oral bisphosphonates is theoretically possible, our collective experience is that in most patients, intravenous therapy has been started because of intolerance or contraindications to oral bisphosphonates. As is normal practice it is important to inform patients of the acute phase response (APR) with intravenous bisphosphonates but perhaps even more so as the symptoms can be mistaken for those of SARS-Cov2 infection. As a pragmatic workaround, we advise that patients should not consider being tested for SARS-Cov2 after intravenous bisphosphonate unless the symptoms persist for more than 4 days.

#### Denosumab

We strongly advise that denosumab treatment delay should not exceed seven months since the last dose [34, 35]. We also urge caution regarding commencement of denosumab unless other options are unsuitable. Where possible a shared care service should be agreed or one-stop clinics established in suitable areas of the hospital with appropriate social distancing measures in place.

#### Anabolic Drugs

For patients who are on the anabolic agents teriparatide or romosozumab, treatment should be continued as these are self-administered injections. If, however, treatment has to be discontinued or patients are coming to the end of their course of treatment it is advised that they transition to oral bisphosphonate in the first instance.

#### Biochemical Investigations

It may be possible to introduce longer intervals between routine blood tests and parenteral treatments such as intravenous bisphosphonates unless there is concern about fluctuating renal function. One option is to have a window of 3–4 months in low-risk patients with eGFR > 40 but to require testing before treatment in a shorter interval of 7–10 days in those with eGFR < 40. Checks of 25(OH)D may not be required in patients who are taking vitamin D.

#### Initiating Treatment in the Absence of DXA

It is possible to advise treatment in the absence of DXA. If this is done there is some evidence that in elderly women with a 10-year fracture risk of > 20% who are prescribed oral bisphosphonates have a reduction in hip fracture risk but not the risk of other fractures [54]. If a decision is made to start treatment DXA should be performed when feasible, fracture risk recalibrated and the need for treatment reviewed. In patients with a recent hip fracture, there is good evidence than zoledronic acid reduces the risk of further fractures [66]. Such evidence is lacking for other treatments.

#### Managing the Hip Fracture Patient

Quality improvement programs can be put in place to increase awareness of bone health assessment among junior doctors and nurse practitioners. This approach has been shown to improve the management of patients with femoral neck fractures as the prescription rates for calcium/vitamin D, bone-sparing drugs and DXA scan requests increased following the implementation of the tool [27]. Access to rehabilitation should be made available with application of the required social distancing and infection control measures. In cases of patients admitted with a hip fracture who are also affected with COVID-19 where mortality has been shown to be high as previously described [26, 44], orthopaedic departments should organise specific accelerated care pathways for their treatment to reduce length of stay in hospital or requirement for intensive care bed [67, 68]. In a recent study of 16 patients admitted with femoral neck fractures and COVID-19 infection, orthopaedic surgery on the day of or within three days of admission contributed to patients' haemodynamic and respiratory stability, improvement in respiratory function and comfort in bed [69].

Bone health review should also include the assessment of spinal deformity, severity and acuteness of back pain, review of chest X-rays and/or CT scans for the opportunistic identification of prevalent vertebral fractures (VFs) with appropriate management offered including pain relief, spinal support [70].

#### Ensuring Patients are not Lost to Follow Up

Recall procedures must be put in place to make sure that patients do not get missed for the administration of osteoporosis drugs and clinical assessment when routine services resume safely in the aftermath of the pandemic when patients have had access to vaccination.

## Conclusions

The screening, diagnosis and management of patients with osteoporosis have proved to be challenging during the COVID-19 pandemic. The follow-up of patients to the metabolic bone clinics has been disrupted which may create future problems due to treatment delays, particularly in patients on denosumab. The quality of osteoporosis care of patients with a new fragility fracture or hip fracture following surgery has dramatically decreased during the pandemic [71].

To avoid the negative impact that the disruption to healthcare services can have on future fracture burden, new guidance about osteoporosis treatment has been issued which involves the design of new care pathways. The new recommendations offer a pragmatic approach to the delivery of osteoporosis services which aim to ensure that the best level of care for fracture prevention is maintained during these unprecedented times. As effective vaccination gets underway and the pandemic is controlled, the alternative models of care instituted such as remote consultations, telehealth medicine, better co-ordination of primary and secondary care, and sharing of resources, should help and incentivise us in building more robust, patient-friendly systems of service delivery.

## Supplementary Information

Below is the link to the electronic supplementary material.

Supplementary file1 (DOCX 26 kb)
