# Supplementary material for: Diagnosis and Management of Osteoporosis During COVID-19: Systematic Review and Practical Guidance
Source: Calcif Tissue Int. 2021 May 18;109(4):351–62. doi: 10.1007/s00223-021-00858-9 (PMC8129963; doi:10.1007/s00223-021-00858-9)
Supplement: Supplementary file 1 — Supplementary file1 (DOCX 26 kb) [file 223_2021_858_MOESM1_ESM.docx]

**Diagnosis and Management of Osteoporosis during Covid-19: systematic review and practical guidance**

G. Hampson ^1,2^ , M. Stone^3^, J. R. Lindsay ^4^, R.K. Crowley^5,6^, S.H Ralston ^7,8^

*^1^ Department of Chemical Pathology and Metabolic Medicine, St Thomas’ Hospital, London, UK,*

*^2^ Metabolic bone clinic, Department of Rheumatology, Guy’s Hospital, London UK*

*^3^ Metabolic bone service, University Hospital Llandough, CF64 2XX*

*^4^ Osteoporosis & Bone Metabolism Service, Musgrave Park Hospital, Belfast, Northern Ireland, UK*

*^5^ Department of Endocrinology St Vincent’s University Hospital, Dublin, Ireland*

*^6^ University College Dublin, Ireland*

*^7^. Centre for Genomic and Experimental Medicine, University of Edinburgh, Western General Hospital Edinburgh EH4 2XU.*

*^8^. Rheumatic Diseases Unit, NHS Lothian Western General Hospital Edinburgh, EH4 2XU,*

**Narratives from five referral centres**

**Belfast**

In Belfast DXA services were downturned at the start of the pandemic for around 3 months, leading to temporary closure of face-to-face fracture liaison clinics, consultant led services and direct access primary care DXA. At an early stage we identified our Denosumab clinic as a high priority area to ensure continuity of treatment delivery, either through existing shared care administration in the community or self-administration. There were around 104 individuals for whom these options where not feasible and who continued to attend face to face clinics for treatment. Clinic capacity was limited to a maximum of 6 review patients, and we deferred new patient starts until there was greater clarity future clinic arrangements. To date we have treated 64 patients via self-administration and 13 of these have confirmed they are willing to continue at present. During the first phase of the pandemic routine access to blood monitoring was not feasible and patients were offered a loading dose of Vitamin D when recent lab data was not available. The benefits of self-administration have included continuation of treatment, with limited impact of treatment delays and a reduction in foot fall to the clinic. While limited support was been needed from the Amgen prolong programme, self-administration has necessitated significant planning and out-reach from the osteoporosis clinical staff and pharmacy team to support blood monitoring and drug dispensing.

We previously had a recognised framework for virtual telephone clinics within the osteoporosis service, supported by clinic templates and booking office arrangements. This system was reactivated at the start of the Pandemic to support patients awaiting osteoporosis review. A small number of patients were offered virtual video consultation on a pilot basis, which was declined on most occasions. The main limitation to starting effective virtual clinic working, was access to DXA, however by early Summer once DXA had reopened, we were able to begin to offer patients direct access DXA via the radiology department, while maintaining close adherence to infection control principles. There was a significant administrative burden for clinical staff who were tasked with reviewing lengthy review lists to determine whether an updated DXA was needed and whether virtual review could be offered to positively impact clinical care. DXA scans were then ordered through the electronic radiology ordering system. Appointments for patients at high risk of Covid with clear contraindications such as pre-existing immunosuppression, chronic chest conditions and the oldest old were deferred temporarily. Our service arrangements have evolved since September with opening of the Belfast drive through phlebotomy hub, which supports diagnostic bloods sampling for new and review patients. This has enabled comprehensive laboratory assessment of new patients and blood monitoring for those initiating or continuing second line agents.

In terms of delivering of 2^nd^ line agents we have managed to continue with Teriparatide initiation using Homecare services with patient education and drug delivery locally. Intravenous Zoledronic acid infusion services reopened in early Summer, which had been prioritised as part of the elderly care frailty unit for hip fracture prevention. During this period there was a significant increase in drug delivery as staff time had been freed from other routine care of elderly duties.

**Cardiff**

In Cardiff we only suspended services for one week in April 2020. We then reinstated all of our clinics: four conventional face to face OP clinics switching to telephone consultations and one pre-existing virtual Denosumab self-injection clinic. We also continued the FLS (Fracture Liaison Service) with smaller numbers of fractures for the first two to three months but quickly returning to normal expected numbers thereafter as lockdown relaxed. The numbers of referrals from primary care to the clinics have been lower but we also introduced an e-advice service at the beginning which has been popular with primary and secondary care physicians which accounts for a proportion of the reduced referrals to the clinics. Face to face consultations restarted in June 2020 for urgent and new referrals where it was clinically justified. The waiting list for my clinics has plummeted to zero for the first time in the twenty nine years I have run the service. However, we have had to run the service with limited access to DXA scans.

In the absence of bone density scans we use FRAX/NOGG with fixed intervention thresholds of 5% for hip fractures and 20% for MOF for those falling in the amber zone (awaiting dashed line for age specific intervention threshold in updated FRAX software) while treating all those over the age of 75 with prior fragility fractures without FRAX or DXA. We can get DXA scans for all of our FLS patients, those on high dose steroids < 65 years old and other high-risk patients. I am now the "authoriser" for DXA scans and rank them based on clinical need.

We have ceased VFA (vertebral fracture assessment) for the time being to increase the number of patients that we can scan. We have provided primary and secondary care colleagues with pragmatic clinical pathways largely based on those discussed above and following the Royal Osteoporosis Society (ROS) guidelines.

In terms of treatment, we mounted an intensive support and explain program by telephone and letter for our patients on parenteral treatment (approximately 2000 per year) in particular targeting those on denosumab. After initial reluctance to attend hospital, after about four weeks we had achieved > 90% attendance rates. We identified a safe treatment area in Llandough Hospital after walking around the hospital to identify clinical facilities that might be appropriate and were no longer being used. The dermatology department had been relocated to another hospital in Cardiff leaving the dermatology treatment centre unused and perfect for socially distanced treatments for four patients at a time with a dedicated outside entrance adjacent to the main Outpatients Department. We did meet with some initial resistance from management but successfully argued that the clinical imperative justified provision of the treatments as discussed above. We also provided the option of "drive-through" injections with covered parking directly outside the department for patients not prepared to attend otherwise.

We already had a denosumab self-injection program whereby patients self-inject and are managed using a virtual clinic and telephone consultations (video available on https://theros.org.uk/healthcare-professionals/covid-19-hub/). This has been expanded and we now have funding in place for one year (from Amgen) to provide a nurse prescriber to enrol the majority of our approximately 1000 patients receiving denosumab. In terms of parenteral treatments, we have maintained our service fully and again reduced our waiting list (remembering fewer new referrals)

So I suppose one of the messages is think on your feet and adapt quickly; be prepared to win over managers who might not be supportive to begin with. Who would have thought that telephone/video consultations would be so successful and acceptable to patients? In many ways our service lends itself well to the digital world and it is something we can embrace and perhaps lead with. So there are real opportunities as a result of Covid as well as undoubted hindrances.

With DXA likely to be limited for some time to come I suppose one might argue that we have to have less reliance on DXA and perhaps a shift to FRAX/NOGG like approaches is now inevitable. Automated software for GPs as exists with Qfracture would be a big advance and is part of the ongoing ROS clinical research program.

**Dublin**

The SVUH osteoporosis service is not conventional - there is no dedicated osteoporosis clinic although there is a huge amount of osteoporosis work. There is a rare bone disease service so some complex osteoporosis cases are reviewed in that unit (cases of atypical femoral fractures or possibility of other underlying disease). Generally osteoporosis care is delivered through 1) inpatient intervention at the time of fracture; 2) FLS-generated DXA assessment which includes FRAX data, dietary calcium intake, wider medical history, falls etc; 3) ISCD-trained physician reporting of FLS and specialty services DXAs which include a treatment recommendation and follow up plan; 4) radiology report primary care DXA referrals which sometimes generate queries to the clinical service – these can be addressed with a letter advice and guidance option or a general endocrinology appointment as appropriate, during the pandemic T Pro video consultations with patients were commenced for this and continue to be used in the complex bone service; 5) there is an allied health professional-led osteoporosis education programme for patients and the public which has moved from in-person lectures and discussion to webinar.

Therapeutic treatment options are different in the Republic of Ireland to the UK – treatments used in primary care can be both suggested and prescribed in the hospital clinic and continued in primary care. Romosozumab is not available to Irish patients. Teriparatide is available only on a hospital prescription and has a nurse education service provided to the patient in their home by the pharmaceutical company, the nurse reports back to the prescribing hospital consultant. Denosumab was administered in primary care (and is very popular with GPs) prior to pandemic, some patients have transitioned to self-administration and will continue. During the summer and latter half of 2021 we were aware of at least 4 patients presenting with back pain who were identified to have new vertebral fractures and who reported delaying a dose of denosumab due between March 2020 and the summer. We managed these with IV zoledronate infusion and analgesia, and offered fitted supportive soft braces to those patients who wished to minimise analgesia use. Patients reported that they were unaware of the risk of delaying denosumab dosing and wished to avoid their primary care centre during the COVID-19 pandemic.

SVUH includes an infusion unit for IV zoledronate which accommodated referrals during the pandemic. Pre-treatment bloods were a bit of an issue at the beginning of the pandemic, but have not been a problem since May / June 2020. Vitamin D loading doses are avoided because of concerns re hypercalciuria; a bone health questionnaire is provided at DXA scanning which allows estimation of dietary calcium therefore vitamin D and calcium supplementation are prescribed on an individual need basis. As part of the hip fracture protocol patients receive zoledronate in hospital and are appointed for another infusion the year after, on discharge. The pandemic has generated new service delivery ideas for osteoporosis including the use of telehealth consultations and webinar education. Future plans that would build on this include the availability of iv zoledronate in the community, much like outpatient antimicrobial use, particularly for those patients seeking to discontinue denosumab; and a combined GP / hospital team clinic to meet the needs of more complex patients in primary care.

**Edinburgh**

During the first wave of the pandemic during March-April 2020 clinic lists were moved from face-to-face appointments to virtual appointments, with the exception of urgent patients. Although NHS Lothian was trying to roll out technologies like NearMe for video consultations we were unable to take advantage of this due to lack of equipment. Numbers of patients attending for DXA scans was drastically reduced to about 20% of the usual numbers due in part to the necessity for social distancing and the reluctance of patients to attend hospital. Urgent DXA scans were still performed. We do not have large numbers of patients on denosumab but that caused major problems since there is no shared care agreement for this medicine in place within NHS Lothian and patients were reluctant to attend hospital for bloods and injections. I personally phoned the GP of every patient receiving denosumab and gained agreement in virtually all cases for the injection to be performed in the community, either at the GP surgery or at the patients home. Staff in the day unit where zoledronic acid infusions are carried out were redeployed to other areas of the hospital and so the waiting list for all infusions increased and we made a decision to delay second and third infusions for periods of between 6-12 months with prioritisation of first infusions for patients commencing treatment. Arranging blood tests during the first wave of the pandemic presented a challenge and this often led to delays in patients starting treatment. This not only applied to parenteral treatments such as zoledronic acid and denosumab but also to oral treatments since we normally advise that renal function and blood calcium are checked prior to introducing osteoporosis treatment. We continued to prescribe teriparatide for severe spinal osteoporosis during the first wave of the pandemic, but numbers were much reduced, due in part to fewer numbers of DXA scans being performed. We very seldom advise treatment to be started without information from DXA unless the patient has been found to have low trauma vertebral fractures. Our advice in this instance has been to start treatment and request DXA so that a quasi-baseline measurement is available for review at a future date.

Unlike some other centres we have not adopted the NOGG based approach of treating without information from DXA since an important part of the discussion with a patient is the likely benefit of treatment and our opinion is that unless there is a vertebral fracture the magnitude of benefit is unknown. This makes it very difficult to counsel patients adequately

Surprisingly the second wave of the pandemic did not have such of an impact on services as the first wave even though numbers of infections were higher. I think that is because NHS Lothian realises that it was not wise to stop treating all other conditions because of Covid. We have therefore adapted and are gradually trying to deal with the waiting list that has grown up over the past year. At present we continue to see new patients face to face before starting parenteral treatments like zoledronic acid and also prior to commencing anabolic treatments like teriparatide and romosozumab which has recently been approved for use by the SMC in patients with severe osteoporosis at high risk of fracture.

**London: Guy’s and St Thomas’ Hospitals**

There was a significant reduction in our osteoporosis services at the start of the pandemic. During the first wave, between March-May 2020, we screened all metabolic bone clinic lists and deferred 41% and 78% of patients in March and April-May respectively for between 3-6 months. The majority of patients were on iv zoledronate, some (about 10%) were either on oral bisphosphonates, HRT or raloxifene and some were at high-risk of Covid-19 infection due to the presence of clinical risk factors and/or co-morbidities (BSR risk scores> 3). Clinic during this time was virtual (telephone consultations) and limited to one list of 6-8 patients who were mostly new referrals or high-risk patients. During this time, we also identified patients on denosumab who were due to attend the hospital for their injection in March-mid-April. They were all contacted and were offered a choice of either attending the hospital for their injection as normal, self-administration or for their GP to administer the drug. Some (40%) chose to attend the hospital as they were local, others (25%) were able to self-administer and were sent the link to the Amgen training video. Their GPs were also contacted if any pre-treatment blood tests or serum calcium monitoring was required. Delivery of the drug to the patients involved a significant amount of organisation and was time-consuming. Some patients (35%) opted for primary care involvement and we contacted their GPs by phone and followed up with a letter. As we did not know how long clinic restrictions would last, we also set up an interim shared care protocol with the local CCGs, although not all CCGs agreed to administer denosumab in primary care. To mitigate risks of treatment delays to patients, particularly those on denosumab, we organised ‘one-stop clinics’ for parenteral treatment. This was set up in line with social distancing and infection control measures. The weekly one-stop clinic has been running since May and we see on average 8-10 patients. Because of logistical issues of obtaining pre-treatment blood tests, patients all have their blood tests on the same day as their appointment and the blood tests (renal and bone profiles) are fast-tracked by the laboratory. Whilst waiting for the results to be available, the patients are seen and assessed by a physiotherapist and given advice about types of exercise to maintain bone health. Because the results of serum 25(OH)vitamin D are not available on the same day, all patients are offered a loading dose of colecalciferol (40, 000 IU) if their vitamin D status was not assessed within the previous 12 months. This new clinic required a significant amount of administrative support to contact and telephone patients reminding them of their appointment. Attendance from June onwards improved (up to 80-90%) but dropped slightly during the second wave early this year. Feedback of this new service has been very positive and we plan to continue and expand it as well as introducing a dedicated denosumab clinic. From the end of May onwards, we opened the clinics again to almost full capacity but all consultations are virtual (via telephone) for the time being. To reduce the number of patients whose treatment was deferred and who are still on the waiting list, we have opened extra pharmacist-led clinics for those on parenteral treatment. We continue to monitor and track this list to make sure we do not miss any patients, particularly those on denosumab. We have also seen an increase in GPs request for advice and guidance via email (5-6/week).

DXA scan services were closed from Mid-March 2020 for six weeks and offered a very limited service from May (1-2 scans/day). They were fully operational from June to December and contacted patients to attend for their DXA scans. A small number of patients declined to attend the hospital and their scans were re-arranged. In the second wave (since mid-January 2021) , we have seen a 50% reduction in DXA scan services as only one scanner was in use but this is being reviewed and the unit may operate at maximum capacity soon. Due to nursing staff deployment, the FLS and case-finding (run by the old people’s assessment unit; OPAU) was suspended during the first wave. During the second wave this time round, a reduced service is being run for patients who are not shielding. The service administrator remained in post and some medical registrars were re-assigned to oversee the infusions. Denosumab has been delayed by four weeks and iv zoledronate by three months in patients who are at medium/high risk. Our administrator remained in post. The FLS database survey nationwide showed that many services were in a similar position.

In summary, we have seen major changes to our service and have had to adapt over the last 12 months. Some of these changes will remain for example conducting more virtual clinics which lends itself well to the management of osteoporosis and reducing the need for face to face appointments, the continuation of the ‘one-stop clinic’ for parenteral agents and the involvement of other health care specialists such as pharmacists in its delivery.
